# A Secondary-Primary Mental Health Integrated Care Model for Communities with Diverse Population and Complex Health Needs – a Case Study with Health Care Utilization Evaluation

**DOI:** 10.5334/ijic.5939

**Published:** 2022-05-16

**Authors:** Clive Bensemann, Irene Suilan Zeng, Helen Hamer

**Affiliations:** 1 Clinical lead of integrated Mental health, New Zealand; 2 Clinical Head of Mental Health of Older People, Division of Mental Health and Addiction, Counties Manukau District Health Board, New Zealand; 3 Division of Mental Health and Addiction, Counties Manukau District Health Board, New Zealand; 4 Department of Biostatistics and Epidemiology, Faculty of Health and Environmental Science, Auckland University of Technology, New Zealand; 5 Former Nurse Lead Integration: Mental Health - Division of Mental Health, and Addiction, Counties Manukau District Health Board, New Zealand; 6 Helen Hamer & Associates Ltd, Auckland, New Zealand; 7 Yale University, USA

**Keywords:** secondary health-care utilization, specialist health-care utilization, integrated mental health care approach, clinical characteristics

## Abstract

Integrated care is expected to improve patient experience, patient outcomes and assist with the increasing demand on health services from those with long term conditions including mental disorder. Few studies have evaluated health care utilization as a consequence of increased integration of mental health care. This study considers the factors known to influence secondary health service utilization and investigated the impact of a locality based mental health integrated model of care (ILoC) providing specialist consultation and liaison advice to primary care, to support early diagnosis and treatment.

Using existing hospital databases, the study-cohort was identified (service users supported by ILoC, and then referred within 6 months to specialist mental health services (MHS) care between 2017– 2018) and compared on health services utilization with a matched-cohort (without ILoC support before referral to specialist services).

The length-of-care in the non-acute MHS was 71% shorter for the ILoC study-cohort, and differences increased in the subgroup taking antidepressants. The ILoC study-cohort was less likely to be admitted to acute MHS on first referral post ILoC intervention and had a 25% lower relative risk of acute MHS admissions at any time in follow-up. There was no difference in the average MHS inpatient length-of-stay. The risk of general hospital acute inpatient admission was marginally higher in the ILoC study-cohort.

**Conclusions:**

ILoC appears to shorten non-acute length-of-specialist-care and reduce acute mental health admission. The study provides a first step in understanding the clinical characteristics and specialist services health-care utilization of patients supported by an integrated mental care approach.

## Introduction

Integrated care is expected to improve patient experience, patient outcomes and assist with the increasing health services demand from those with long term conditions, including mental health needs [1,2,3,4,5,6,7,8]. Models of usual care for mental health service users typically involve a separation between specialist care and primary care services. Service users usually access specialist MHS via formal referral from primary care, through presentation to MHS crisis teams often based in the community, or through the hospital Emergency department. Specialist care is required for the small proportion of the population with severe acute or enduring disorders. An integrated care approach provides earlier access to specialist mental health support for those with severe disorder but also potentially early support to a wider population with less severe disorder.

Integration and collaborative care are terms often used together in the literature. A Cochrane review [9] of 79 randomized controlled trials comparing collaborative care with usual care in 24,308 participants with depression and anxiety demonstrated that collaborative care increased the number of medications used and led to improvements in mental health-related quality of life. A second Cochrane review [10] of collaborative care for severe mental illness found only one study which met their inclusion criteria [11]. This included 306 participants with bipolar affective disorder and reported a lower risk of psychiatric hospital admission in the collaborative care group at years 2 and 3 follow-up. No difference in the treatment cost was found.

Other literature describes a range of impacts of integration initiatives on health service utilization including a decrease in non-acute service utilization and an increase in acute general hospital services utilization [12,13,14].

## Development and implementation of ILoC

Counties Manukau Health (CMH) is a district health board in South Auckland New Zealand and serves a multi-ethnic community including Maaori, Pacific and Asian communities with significant socio-economic deprivation [15].

CMH aims to deliver the triple aims of improved quality, safety and patient experience of care, improved health and equity for all populations, and better value [15,16].

A locality based integrated care model (Integrated Locality Care (ILoC)) was implemented in late 2016 as one component of a wider ‘whole of system’ change in the mental health services aiming to make specialist services support accessible to a larger part of the CMH population [15,16].

An earlier collaborative project in the service had changed all position descriptions from ‘case manager’ to ‘mental health clinician in scope’ aiming to reposition the specialist workforce to be ‘facing’ primary care, to build stronger relationships with primary care and so to develop new responses to the challenges facing access to specialist mental health systems in Aotearoa – New Zealand [17,18,19].

The ILoC implementation expected that the specialists services working closely with primary care would, in time, shift specialist staff focus to integration and adapt their practice to greater collaboration and ‘sharing the care’ with primary care partners [20]. ILoC focused primarily on a consultation liaison approach, aiming to build capacity and capability in their primary and community partners, and to intervene early, keep people well, and support service user’s ability to self-manage their heath needs. These changes are significantly different from the usual care delivered particularly by secondary adult specialist services to a small proportion of the mental health population.

ILoC activities include telephone and face-to-face consultation with primary care providers; some one-off patient assessments or joint sessions in a GP practice, a marae clinic-, (the traditional meeting ground for a Maaori community) or with a school counsellor; some brief therapeutic interventions, and the ‘signposting’ of relevant alternative community-based resources to providers and service users. Contact for liaison support were made by ‘request’, rather than formal referral, and the primary care clinical record is shared and used by all parties. Care delivery remains substantially with the primary care practitioner (general practice, school health services, aged residential care providers (ARC), or marae-based clinics).

This service is provided by a multidisciplinary team including Psychiatrists, Occupational therapists, Registered nurses and Social workers, Community Support workers (non-government-organization (NGO)) staff and Community Alcohol and Drug Service (CADS) clinicians. ILoC currently serves approximately 30% of general practices within the CMH area, as well as 10 high schools, 20 aged residential care providers (ARC) and one marae-based clinic. Clinical psychologists also provided clinical supervision to some ILoC teams.

One typical scenario of an ILoC request is from a school nurse requiring guidance on a student. After verbal consultation and a brief assessment with an ILoC clinician, the student was referred to the non-acute mental health service. If this was usual care, the student will need to see a GP first and wait for GP to refer them to specialist secondary care. If the student’s condition was assessed to be acute, they would be referred immediately to the crisis mental health team.

We conducted a matched-cohort observational study to investigate the impact of ILoC on secondary-care utilization, including specialist mental health community care service units and inpatient hospital care services and to identify other factors that affected health services utilization.

**Figure 1:**
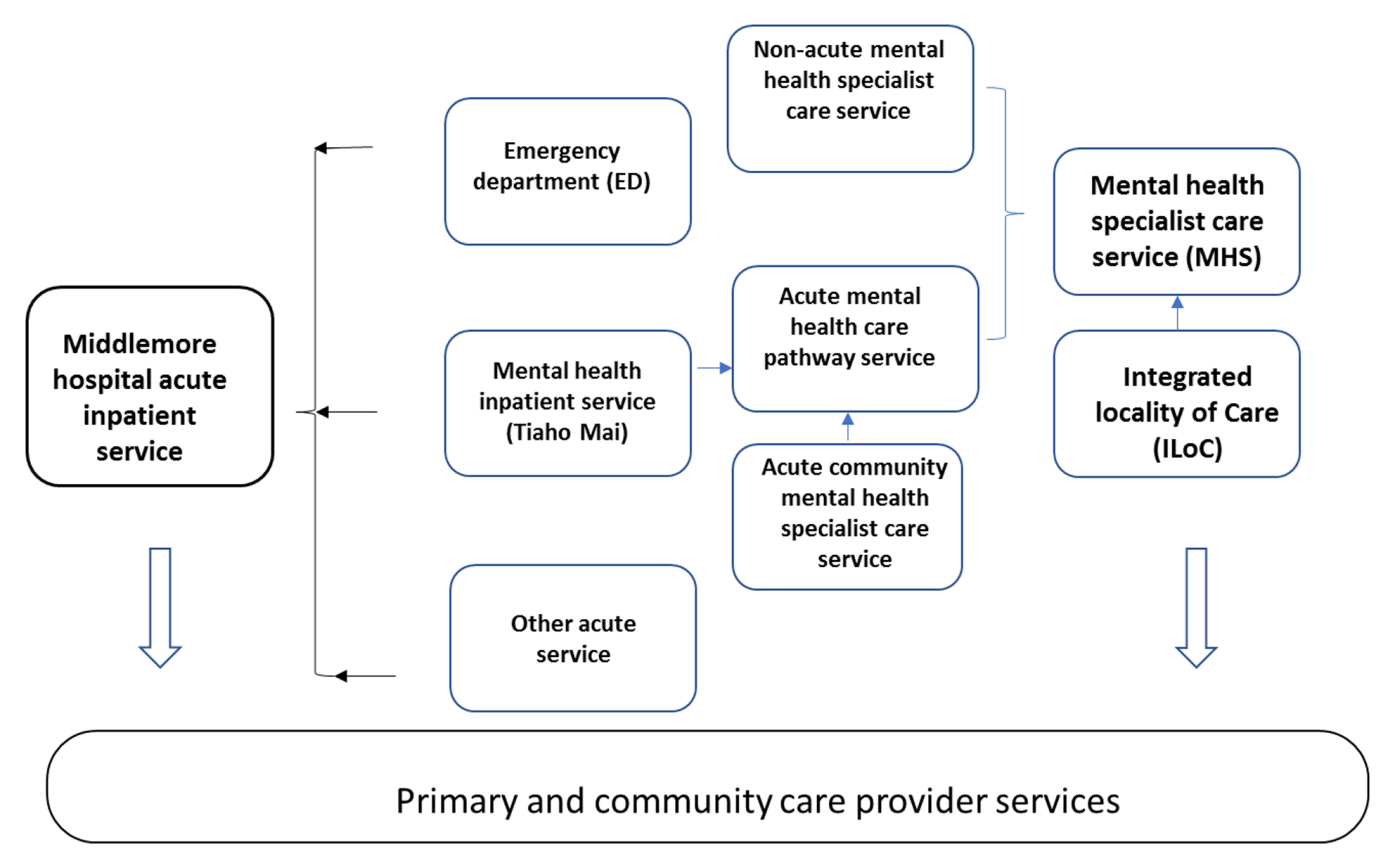
The operational structure of Mental health specialist services and Emergency department.

CMH specialist mental health services (MHS) provides both acute care pathway service (acute-MHS) and non-acute ‘integrated’ specialist care service (non-acute MHS). The acute pathway includes the mental health inpatient unit and acute community mental health specialist care service (Figure 1).

The study used evidence from the literature to inform the design, including identifying other factors known to associate with the utilization of mental health and inpatient hospital services.

Factors affecting utilization of unscheduled hospital admission for mental health service users, include service users’ demographics [21,22], social functioning [21,23,24,25], comorbidities [21,25], mental health morbidities [13] and suicidal behaviours [14,26]. In studies of care cost, Siskind, Harris [13] identified diagnosis of psychosis as the strongest predictor for the total acute mental-health care cost 1-year after the patient had an acute public psychiatric hospital admission; illness acuity and prior acute psychiatric inpatient admission also predicted higher costs. A study including 28,716 ED visits [27] examined hospitalization within those patients discharged from ED with behavioural health conditions.Differences in likelihood of hospitalization were associated with gender, race, health insurance status, mode of ED arrival, and behavioural health diagnosis.

Systematic reviews of primary care features identified that proximity influenced patterns of unscheduled secondary care use [28]. We used Andersen’ behaviours model [29,30,31] to describe and classify these multiple factors into predisposing factor, enabling factor and clinical factors in the current study.

## Evaluation

### Study design

We used a quantitative approach, conducted a retrospective cohort study with a matched cohort.

*The ILoC primary-cohort*: service users of CMH exposed to an ILoC intervention through their primary care/school health providers between Jan 2017 and June 2018.

*The ILoC study-cohort*: a subset of the primary-cohort only included those who were referred to CMH mental health specialist care (MHS) within 6 months.

*The matched -cohort*: selected from those who were referred to MHS during the same period but without receiving ILoC support, matched by demographics and referral month.

Each participant was followed for a period of 6 months – 1 year from the date of their referral to MHS.

The study was approved by the CMH locality and New Zealand Health and Disability Ethics Committee (19/CEN/41/AM01).

### Linked databases and study setting (outcomes, confounders, and other factors)

**Figure 2:**
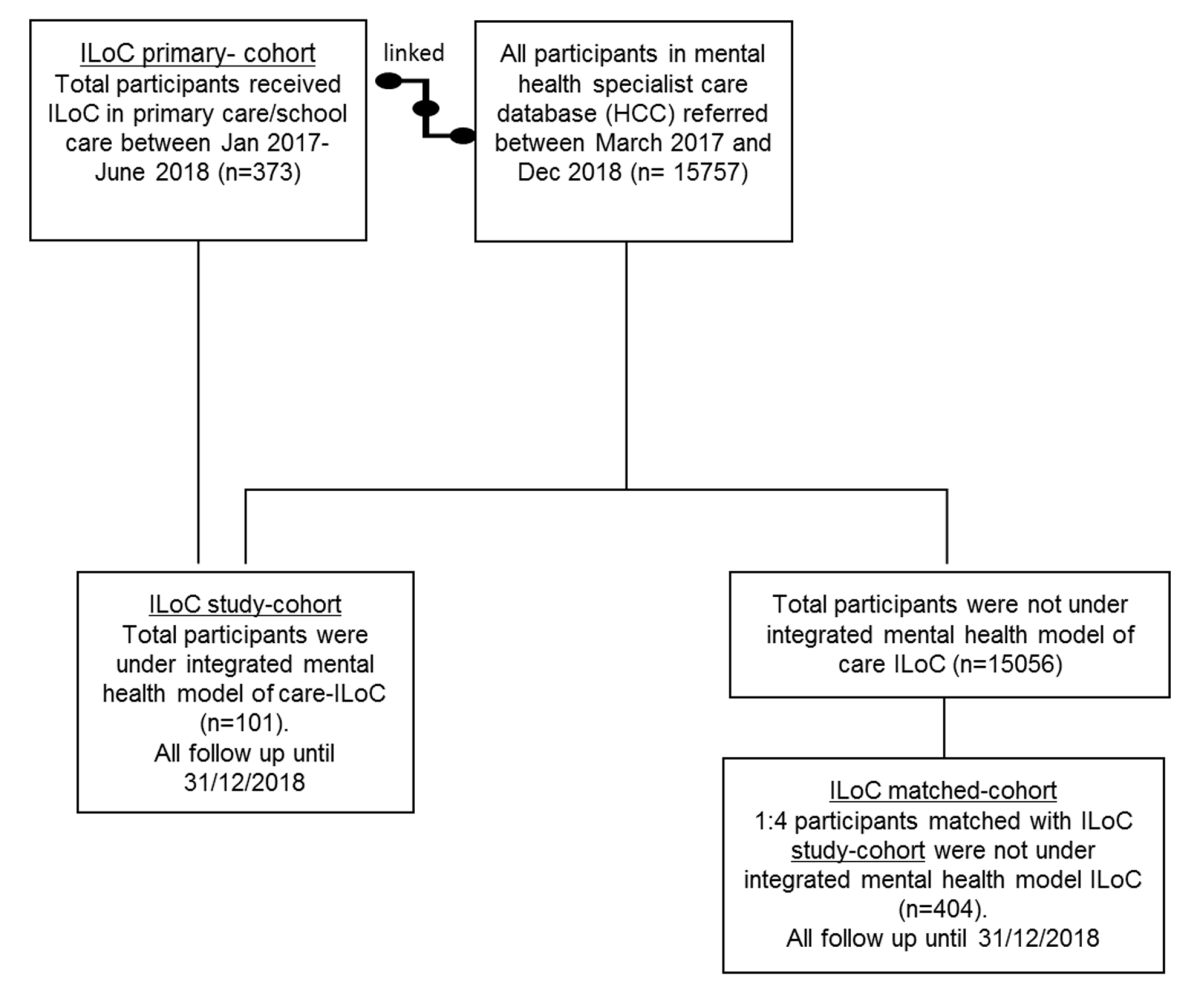
The study participants flow chart.

Using the information and data linkage from two major Counties Manukau Health databases HCC and iPIM, the ILoC primary-cohort and the ILoC study-cohort were identified. The ILoC study and the matched-cohort were selected from the same referral database, in the same period using a ratio 1:4 between ILoC study and matched-cohorts (Figure 2). These were service users without any significant connection to the ILoC service but in the same referral population to mental health specialist care (MHS). The cohort was matched by age group, gender, ethnicity, month, and year of referral.

The primary outcomes assessed were non-acute MHS length-of-care, admission/readmission to acute MHS and acute admission from the first MHS referral. The secondary outcomes were length-of-care in acute MHS, MHS inpatient length-of-stay, and general hospital inpatient admission.

For general hospital inpatient admission, we extracted the hospital inpatient admission records, one-year before the earliest first referral and all records post first MHS referral in the study period. We then reviewed the reasons for inpatient admission to identify those who were admitted during follow-up for suicide attempts, those admitted for any other reasons associated with a mental health condition, and those admitted for other non-mental health reasons.

Based on the literature described in 2.0, we included predisposing factors (age, gender, ethnicity, country of birth), enabling factors (distance to the hospital), clinical needs factors (using medications prescribed after the first referral as a proxy), socioeconomic status, previous self-harm or suicidal attempts, and country of birth. Prescribed medication, classified as benztropine, antidepressant, antipsychotic, hypnotic, mood stabilization and anxiolytic, was used as a proxy for mental health condition and clinical need indicator.

The collated information of both cohorts included: referral to and discharge dates from mental health specialist (MHS) service, referral reasons and referral sources, previous number of admissions to MHS, hospital inpatient admission diagnoses, other MHS service referral information, inpatient admission dates, reasons for admission and demographics.

### Statistical analysis

A pilot study using an ILoC subset and its unmatched-cohort was used to investigate the concept and feasibility. Based on the pilot and sample size estimation, a matched-control by ethnicity, age group and referral time was proposed to eliminate these confounding effects and enhance the statistical power.

The outcomes of non-acute or acute MHS length-of-care, and inpatient MHS length-of-stay were analysed using the mixed effect model to account for the repeated mental health referral episodes and were adjusted for confounding effects (proximity-distance to the hospital, country of birth, deprivation score, psychotropic medication), matched factors (age group, gender, ethnicity) and the exposure factor (ILoC intervention). The likelihood of an acute first MHS admission was compared between the ILoC study and ILoC matched-cohort using a multiple logistic regression. Length-of-stay and length-of-care were log-transformed in the mix effect models.

The other outcomes (admissions to the acute MHS, any general hospital inpatient admission) were compared between the ILoC study and matched-cohort using the Cox regression model with a robust variance to account for the re-occurring admissions and adjusted by the same abovementioned confounding effects, matched factors and the exposure factor.

Pre-specified subgroup analyses were conducted for those prescribed antidepressants and antipsychotics after the first referral. Known confounders are all included in the models.

In the result, p value < 0.05 was considered to be statistically significant. Outcome estimates were all provided with their 95% confidence intervals. SAS institute released software version 9.4 and R version 4.0.0 were used in the analysis.

### Results

#### Demographics

Between Jan 2017-June 2018, 373 service users received ILoC intervention through primary care providers (GP, school counsellor, aged residential care, or other community services). Of the 373 service users, 101 had a referral to specialist MHS within the following 6 months. A matched-cohort of 404 service users was selected from the same MHS referral population in the same period. As shown in Table 1, the mean ages of these three cohorts (ILoC primary, ILoC study and matched-cohorts) were 48 (SD 29), 41 (SD 28) and 41 (SD 28) respectively. These three cohorts had similar age-group distributions. The ILoC study-cohort had a higher percentage of females (65.4%) compared to the ILoC primary-cohort (58.5%). New Zealand European and Maaori were the two largest ethnic groups in the three cohorts. Most of the service user participants were born in New Zealand and country of birth was evenly distributed across the three cohorts. The ILoC primary-cohort and study-cohort lived at significantly greater distance from the hospital than the matched-cohort.

**Table 1:** Demographics of the three cohorts.


	PRIMARY ILOC COHORT	ILOC STUDY-COHORT	MATCHED-COHORT	P VALUE OF COMPARING ILOC STUDY VS. MATCHED-COHORTS

	n = 373	n = 101	n = 404	

Age (mean (std))	48 (29)	41 (28)	41 (26)	0.86†

**Age groups**				

0–19	113 (30.3%)	37 (36.6%)	148 (36.6%)	

20–29	33 (8.9%)	9 (8.9%)	36 (8.9%)	

30–39	24 (6.4%)	8 (7.9%)	36 (8.9%)	

40–49	27 (7.2%)	9 (8.9%)	32 (7.9%)	

50–64	43 (11.5%)	11 (10.9%)	44 (10.9%)	

65 and over	133 (35.7%)	27 (26.7%)	108 (26.7%)	

**Gender**				

Female	218 (58.5%)	66 (65.4%)	269 (66.7%)	

Male	155 (41.6%)	35 (34.7%)	135 (33.4%)	0.81§

**Ethnicity**				

Asian	25 (6.7%)	9 (8.9%)	16 (3.9%)	

European	281 (75.3%)	69 (68.3%)	295 (73.0%)	

Maaori	53 (14.2%)	20 (19.8%)	85 (21.0%)	

Other	8 (2.1%)	1 (1.0%)	0 (0%)	

Pacific	6 (1.6%)	2 (2.0%)	8 (2.0%)	0.10§

**Born in New Zealand**				

No	75 (20.1%)	19 (18.8%)	50 (12.4%)	

Unknown	13 (3.5%)	0	11 (2.7%)	

Yes	285 (76.4%)	82 (81.2%)	343 (84.9%)	0.47§

**Deprivation score**				

**mean (std)**	5 (3)	5 (3)	6 (3)	0.17$

**Distance (km) to hospital (median (IQR))**	24.6 (9.6, 29.4)	22.7 (9.4, 29.4)	9.6 (6.4, 14.6)	<0.0001#


† Analysis of variance. # Kolmogorov-Smirnov two sample tests (empirical distribution test). § Chi square test or fisher exact test.

#### Clinical profile

The ILoC study-cohort and matched-cohort had similar pre-referral hospital inpatient admission rates (43/101 (43%) vs. 54/404 (38%), p = 0.48), and a similar proportion of previous suicide attempts (6/101 (5.9%) vs. 14/404 (3.5%), p = 0.39). The ILoC study-cohort had a significant higher proportion of general hospital inpatient admission for reasons related to mental health than the matched-cohort before first referral to MHS (9/101 (8.9%), 12/404 (3.0%), p = 0.01), but there were no significant differences in the admissions due to “any reason” between the two cohorts (37 (36.6%) vs. 116 (28.7%), p = 0.12). A comparison in cohorts’ medication profiles after the first referral (including continuing medications) to MHS (Table 2) shows that, psychotropic medications were more commonly prescribed in the ILoC study-cohort than the matched-cohort. Antidepressant and antipsychotic medication were the major prescribed psychotropic medications in both cohorts. Antidepressant had a similar prescription proportion; antipsychotic had a 7% higher prescription frequency in the ILoC study-cohort. The other commonly prescribed psychotropic medications in both cohorts were anxiolytics, hypnotics, and mood stabilizers. Anxiolytic and mood stabilizer medications had significantly higher prescription proportion in the study-cohort than the matched-cohort.

**Table 2:** Clinical profile of study-cohort and matched-cohort.


	ILOC STUDY-COHORT	MATCHED-COHORt	P VALUE#

Referral for first specialist episode was from GP	47 (46.5%)	184 (45.5%)	0.86

	N = 101	N = 404	

Medication profile post first referral			

ADHD	0 (0%)	9 (6.9%)	

Benztropine	2 (2.0%)	3 (2.3%)	0.52

Antidepressant	22 (21.8%)	77 (19.1%)	0.53

Antipsychotic	25 (24.8%)	72 (17.8%)	0.11

Hypnotic	12 (11.9%)	33 (8.2%)	0.24

Mood stabilization	8 (7.9%)	13 (3.2%)	0.03*

Anxiolytic	14 (13.9%)	30 (7.4%)	0.04*

Previous suicide attempts 1 year before Jan 2017	6 (5.9%)	14 (3.5%)	0.39

Have previous ED and inpatient wards admission	43 (42.6%)	154 (38.1%)	0.48


# Chi square or fisher exact test.

#### Primary outcomes

##### Length-of-care in non-acute MHS

The length-of-care in non-acute community MHS was the period a service user was under care of the community MHS specialist teams.

The episodes of admission to MHS during the study period are compared in Table 3. The median non-acute MHS length-of-care was 66 days for the ILoC study-cohort and 76 days of the matched-cohort.

**Table 3:** MH&A Length of care/stay.

DATA PRESENTED ARE DAYS	ILOC STUDY-COHORT	MATCHED-COHORT

	**no of admission episodes = 69**	**no of admission episodes = 326**

observed acute service length of care in days median (IQR)	5 (1–10)	5 (2–11)

	**no of admission episodes = 26**	**no of admission episodes = 52**

observed inpatients length of stay median (IQR)	13 (5–26)	12 (5.5–22.5)

	**no of admission episodes = 123**	**no of admission episodes = 362**

observed Non acute length of care median (IQR)	66 (8–205)	76 (18–189)

	**no of admission episodes = 218**	**no of admission episodes = 740**

observed all length of care in days (median (IQR))	14 (3–99)	13 (4–76)


After controlling for the confounding effects and covariates, the predicted average length-of-care in non-acute MHS was 55 days for the ILoC study-cohort and 76 days for the matched-cohort. The length-of-care in the ILoC cohort was therefore about 71% of the length-of-care in matched-cohort (95% confidence interval (CI) of the ratio: 0.47 to 1.08, p = 0.11). Service users in both the study and matched-cohorts who were prescribed antidepressants at any time during the study period (used here as a proxy for diagnosis/clinical need) had on average a significant 67% longer length-of-care in non-acute MHS than those not prescribed antidepressants (ratio: 1.7, 95% CI: 1.1 to 2.7, p = 0.03) (Table 4).

**Table 4:** Length- of- care (days) in non-acute MHS – comparison between ILoC study and matched-cohort adjusted for confounding factors.


	ESTIMATED RATIO IN DAYS	CONFIDENCE INTERVAL	P VALUE

**study ILoC cohort vs. matched-cohort**	0.71	0.47–1.08	0.11

***Predisposing factors***			

Age at referral (each year increase)	0.99	0.98–1.00	0.04*

ethnicity			

Asian and others vs. European	1.13	0.46–2.78	0.79

Maaori and Pacific vs. European	1.04	0.64–1.69	0.86

Gender			

Female vs. Male	1.09	0.75–1.57	0.66

Born in NZ (Yes vs. No)	0.95	0.48–1.88	0.89

***Enabling factors***			

deprivation index	1.03	0.97–1.11	0.31

***Clinical needs factors***			

Antidepressant (Yes vs. No)	1.67	1.06–2.65	0.03*

Antipsychotic (Yes vs. No)	1.31	0.78–2.20	0.30

Hypnotic (Yes vs. No)	0.62	0.30–1.31	0.21

mood stabilization (Yes vs. No)	0.90	0.41–1.95	0.78

anxiolytic (Yes vs. No)	1.75	0.80–3.82	0.16


A subgroup analysis including only those prescribed antidepressants showed that the ILoC study-cohort had 28.6% shorter non-acute length-of-care than the matched- cohort (with a 95% CI: 12.1% –67.6%, p = 0.005).

##### Admissions to acute-MHS (including inpatient and other acute mental health specialist care)

The ILoC study-cohort had a lower risk of acute MHS admission at any time during the follow-up period, than the matched-cohort (hazard ratio: 0.75, 95% CI: 0.54 to 1.03, p = 0.08), with a marginal statistical significance. All service users had significantly higher risk of an acute MHS admission if they were prescribed antipsychotics medication, or antidepressants or if they live in regions with higher deprivation index (Table 5).

**Table 5:** Admissions to acute MHS -comparison between ILoC cohort and matched-cohort adjusted for confounding factors.


	HAZARD RATIO	95% CONFIDENCE INTERVAL	P VALUE

Study group (study ILoC cohort vs. matched-cohort)	0.75	0.54 – 1.03	0.08

Age at referral	1.00	1.00 – 1.01	0.34

**ethnicity**			

Asian and Other vs. European	1.18	0.66 – 2.12	0.58

Māori and Pacific vs. European	1.14	0.84 – 1.56	0.40

Gender			

Female vs. Male	1.13	0.85 – 1.50	0.42

Born in NZ (No verse Yes)	0.77	0.47 – 1.26	0.30

Deprivation index	1.10	1.05 – 1.15	<0.0001*

antidepressant (Yes vs. No)	1.48	1.11 – 1.98	0.007*

Antipsychotic (Yes vs. No)	1.61	1.19 – 2.17	0.002*


Subgroup analyses were conducted for service users who were prescribed antidepressants and those prescribed antipsychotics. Service users prescribed antidepressants in the ILoC study-cohort, had marginally lower risk of acute MHS admission than those in the matched-cohort (hazard ratio: 0.65, 95% CI: 0.40–1.07, p = 0.09). There was no significant difference in the risk of admission to acute MHS between service users prescribed antipsychotics in the two cohorts (hazard ratio: 0.87, 95% CI: 0.55–1.37, p = 0.54).

##### Acute admission in the first MHS episode after referral

The ILoC study-cohort was significantly less likely to have an acute MHS admission immediately post referral when compared to their matched-cohort (34.7% vs. 49.3%, Table 6). This difference increased after adjusting for confounders and covariates (odds ratio of 0.52, 95% CI: 0.32–0.85, p = 0.009) (Table 7).

**Table 6:** First MHS episode after ILoC intervention in ILoC study-cohort compared to matched-cohort.


	ILOC STUDY-COHORT	ILOC MATCHED-COHORT	P VALUE
		
	Count (%)	Count (%)	

	n = 101‡	n = 404‡	

Acute admission	35 (34.7%)	199 (49.3%)	

Inpatient admission	6 (5.9%)	21 (5.2%)	

Non-acute admission	60 (59.4%)	184 (45.5%)	0.03*


‡ Number of participants.

**Table 7:** Acute admission in the first MHS episode after ILoC intervention – adjusted for confounding factors.


	ODDS RATIO	CONFIDENCE INTERVAL		P VALUE

study ILoC cohort vs. matched-cohort	0.52	0.32	0.85	0.009*

***Predisposing factors***				

Age at referral (each year increase)	1.01	1.002	1.02	0.01*

**ethnicity**				

**Asian and others vs. European**	1.40	0.55	3.53	0.48

**Maaori and Pacific vs. European**	1.69	1.01	2.83	0.05*

**Gender**				

**Female vs. Male**	1.74	1.15	2.61	0.008*

**Born in NZ (Yes vs. No)**	1.48	0.77	2.87	0.24

***Enabling factors***				

**deprivation index**	1.16	1.09	1.25	<.0001*

***Clinical needs factors***				

**Antidepressant (Yes vs. No)**	1.69	1.03	2.77	0.04*

**Antipsychotic (Yes vs. No)**	2.54	1.52	4.26	0.0004*


The two cohorts had similar proportions of service users with an acute mental health inpatient admission in their first MHS episode (ILoC study-cohort 6 (5.9%) and matched-cohort 21 (5.2%)) (Table 6).

Service users with Maaori or Pacific prioritized ethnicities, female service users, service users living in the high deprivation index areas, service users prescribed antidepressants, and service users prescribed antipsychotics were all more likely to be admitted to the acute MHS in their first MHS episodes (Table 7).

In service users prescribed antidepressants, the likelihood of an acute MHS admission in their first referral was significantly lower in the ILoC cohort than the matched-cohort (odds ratio: 0.30, 95% CI: 0.12–0.75, p = 0.01). In service users prescribed antipsychotics, the likelihood was also lower in the ILoC cohort (odds ratio: 0.38, 95% CI: 0.15 – 0.96, p = 0.04).

#### Other outcomes

##### General hospital inpatient admissions (excluding mental health inpatient services) during follow-up

Of all service users in the ILoC study and matched-cohort who had a general hospital inpatient admission after their first MHS referral during follow-up period, only 1 from the ILoC study-cohort (1%) and 5 from the matched-cohort (1.2%) were admitted for suicide attempts. There was a marginal difference in the proportion of people who had at least 1 general hospital inpatient admission due to mental health reasons or self-harm between the two cohorts (10/101 (9.9%) vs. 20/404 (5.0%, p = 0.06), and no significant differences in admissions for any reason (physical or mental health) (29 (28.7%) vs. 93 (23.0%), p = 0.23).

After controlling for the confounding effects and covariates, the ILoC study-cohort had an overall 1.60–fold higher risk (95% CI of hazard ratio: 0.96 to 2.68, p = 0.07) of general hospital admission for any reasons post referral than the matched-cohort. The other significant contributing factors associated with a higher risk of general hospital admission were older age, higher derivation index and shorter distance-to-hospital.

##### Length- of- care in acute-MHS and MHS inpatient length-of-stay

The median acute-MHS length-of-care was 5 days for the ILoC study-cohort and 5 days for the matched-cohort, and 13 days versus 12 days for inpatient length of stay (Table 3). There were no significant differences in acute- MHS length-of-care and MHS inpatient length-of-stay between the two cohorts after adjusting for confounding factors. Prescription of antipsychotics, a reflection of clinical need, was significantly associated with prolonged MHS inpatient length-of-stay (hazard ratio 3.43, 95% CI 1.56–7.56, p = 0.004).

### Discussion

Service users supported by Integrated locality care (ILoC) before referral to specialist mental health services (the ILoC study-cohort) had higher frequency of antidepressant, antipsychotic, hypnotic, and anxiolytic prescription, compared to those not referred on. This ILoC study group also appeared to have a higher risk of general hospital inpatient admission (excluding mental health inpatient services), compared to service users with a similar demographic profile who had not received ILoC support, before being referred to mental health specialist. The ILoC study group had shorter periods of care in the non-acute mental health services and were less likely to be admitted acutely. These results are consistent with the findings of the 2 Cochrane reviews.

Archer’s Cochrane review and meta-analysis [9] of endpoint-antidepressant medication use in 44 studies (10,117 participants) reported a result in favour of the collaborative care with risk ratios (RRs) of 1.47, 1.43, and 1.22 at 0–6 months, 6–12 months and 13–24 months follow-up respectively. Although medication prescription is not an outcome measure in our study, the ILoC study-cohort had higher prescription proportion than the matched-cohort in psychotropic medications prescription and had significantly higher proportions of mood stabilization and anxiolytic medication prescription after the first referral.

Reilly’s review [10] reported a lower risk of psychiatric hospital admission in the collaborative care group at years 2 and 3 follow-up, with a risk ratio of 0.75 (95% CI: 0.57, 0.99), and 0.73 (95% CI: 0.53, 1.01), respectively. This review included studies with low grade evidence using the Cochrane collaboration’s tool for assessing risk of bias and the reviewers advocate for more high-quality studies in the future to assess the effectiveness of collaborative care models.

Our study compared mental health re-admission within 6 months after the first specialist care referral (following ILoC intervention) and identified different patterns in secondary care utilizations. Consistent with Bauers’ finding [11], the ILoC study-cohort had lower risk of acute mental health admission (hazard ratio of 0.75, 95% CI: 0.54, 1.03), although they had a marginally significant higher general hospital inpatient admission rate (for mental health or physical health reason) with a hazard ratio of 1.6 (95% CI: 0.96–2.68). Our study follow-up time was less than 2 years, and the study-cohort was different in severity of mental health condition compared to the Bauer’s study [11] which only included participants with serious mental illness (SMI) defined as schizophrenia, schizophrenia like disorder, or bipolar disorder.

Busse and Stahl [32] evaluated integrated care interventions for people with a range of chronic conditions in three European Countries; and examined mortality, use of hospital care, process indicators, patient and provider experience, and cost per patient-year. The evaluations showed mixed results. Two countries (England, Netherlands, and Germany) demonstrated increases in hospital admissions or hospital emergency admissions. England’s 16 integrated care pilots showed decreased elective admissions and outpatient admissions, and the Netherlands bundled payment system demonstrated decreased specialist care. The German Gesundes Kinzigtal integrated mental health program reported a decrease in the hospital length-of-stay. Comparatively, our results are consistent with those from this latter integrated model in the utilization of hospital care i.e., decreased length of specialist care and increased general hospital admission. The German integrated care program, which targeted about 50% of population in Kinzigtal region is a good example worth further studies.

A comparison of the ILoC model to the six elements of the chronic care model (CCM) [33,34] in primary care practice and the triple aims, provides an opportunity to target areas for improvement. Four of the six CCM elements (1. increasing providers’ expertise and skills; 2. educating and supporting patients; 3. making care delivery more team-based and planned; 4. making better use of registry-based information systems) were proved to lead to great improvement in health outcomes [33,35]. The ILoC model addresses 1 and 3 of these domains. It also targets health equity by increasing accessibilities to primary/community care-provider partners. A third element is addressed by ‘Wellness support’ which is another initiative of the wider CMH integration changes which targeted improved quality, safety, and patient experience of care. Wellness support seeks to address ‘educating and supporting patients’ by providing primary care practitioners with a wider set of self-management resources for patients [36]. The fourth element, use of registry-based information systems is challenging because of the separately configured clinical information systems in each of the primary care and specialist care organisations. ILoC has succeeded in using the existing systems to produce a shared record specifically of ILoC support in both the primary care and secondary care clinical records. However, the use of shared registers should be considered as part of future development.

There was insufficient data to allow separate analysis by ethnicity in this study. Further adaptions to the ILoC integration approach should specifically consider the needs of the Maaori and Pacific communities who together make up about half of the CMH populations. These communities experience significant inequity in mental health outcomes. Models such as Durie’s Te Whare Tapa Wha Maaori health approach could inform this work [37,38].

#### Predictors to health care utilization

Huntley, Lasserson [28] completed a systematic review of 48 studies, and identified patient factors associated with emergency department hospital admissions, which included increased age, reduced socioeconomic status, lower educational attainment, chronic disease, and multi-morbidity. Practice features included the patient proximity to general practice compared to hospital. Of these, one of the US studies showed that proximity to a primary health care practice reduced ED attendance, but the proximity to a hospital increased utilization of ED. Deprivation index was a significant factor positively contributing to ED attendance in 4 studies (one reported RR 1.42). Our study found a similar significant result in proximity and deprivation index. The hazard ratio of general hospital admission from one unit increase in deprivation index is 1.07, and a hazard ratio from one kilometre increase in the distance- to- hospital is 0.98.

Our finding of the significant association between prescription of antipsychotic medication and inpatient mental health length-of-stay was also consistent with findings of Siskind et al.‘s [13] study. In this study, patients with psychotic disorder have 3.34 times greater number of acute psychiatric inpatient bed-days in the year after discharge and double the total acute mental health costs, compared to patients with other diagnoses.

#### Lessons learned

- This study is the first to investigate the secondary care utilization of an integrated mental health care model using a matched cohort design. It has completed data in primary and secondary outcomes; it used two major databases to provide information and assessed the outcomes independently. However, the study was retrospective and relied on the available information from the administrative databases. We cannot use a parallel control group to compare with the intervention group as would occur in a randomized control trial. There were limits to the available information we can use. For example, we cannot provide accurate information for analysis of living conditions. We have used prescription of medication as a proxy for mental health condition/diagnosis. Prescription of medication was also shown to be a potential mediator for length of care. These phenomena made interpretation more difficult.
- The ILoC approach should implement further changes consistent with the six elements of the CCM model, and with a holistic Maaori health model such as Te Whare Tapa Wha. Qualitative and quantitative method can be used in evaluation and research to assess the impact of any such changes. In particular, future studies must have sufficient participants to consider the effects of integration on the outcomes for the Maaori and Pacific population.
- A future study should focus on a larger cohort with longer prospective follow-up of patient outcomes post ILoC intervention and acquire information (e.g., from primary/community care, patient interview) that is not currently available in the existing databases.

### Conclusion

The study provides a first step in understanding the clinical characteristics and health-care utilization of patients with mental health needs supported by an integrated locality care model. ILoC appears to shorten non-acute length- of-specialist care and reduce acute mental health admission. Future application includes continued improvements in the integrated system and the model of care. These need to reflect the clinical needs, and other factors influencing health service utilization identified from the study.

## References

[1] Behavioral Health Integration Report and Recommendations. The Robert Bree Collaborative; 2017.

[2] Coventry P, Lovell K, Dickens J, Bower P, Graham CC, McElvenny D, et al. Integrated primary care for patients with mental and physical multimorbidity: cluster randomised controlled trial of collaborative care for patients with depression comorbid with diabetes or cardiovascular disease. BMJ. 2015; 350. DOI: 10.1136/bmj.h638 PMC4353275 25687344

[3] Foundation TKF. Integrating Physical and Behavioral Health Care: Promising Medicaid Models Medicaid; 2014.

[4] Goodrich DE, Kilbourne AM, Nord KM, Bauer MS. Mental Health Collaborative Care and Its Role in Primary Care Settings. Curr Psychiatry Rep. 2013; 15(8). DOI: 10.1007/s11920-013-0383-2 PMC3759986 23881714

[5] Kroenke K, Unutzer J. Closing the False Divide: Sustainable Approaches to Integrating Mental Health Services into Primary Care. J Gen Intern Med; 2017. DOI: 10.1007/s11606-016-3967-9 PMC5377893 28243873

[6] Mental Health and Integration. The Economist Intelligence Unit Limited; 2014.

[7] Naylor C, Das P, Ross S, Honeyman M, Thompson J, Gilburt H. Bringing together physical and mental health: A new frontier for integrated care; 2016. 10.1177/0141076816665270 PMC5066536 27729592

[8] Patel V, Belkin GS, Chockalingam A, Cooper J, Saxena S, Unutzer J. Grand Challenges: Integrating Mental Health Services into Priority Health Care Platforms. PLOS Medicine. 2013; 10(5). DOI: 10.1371/journal.pmed.1001448 PMC3666874 23737736

[9] Archer J, Bower P, Gilbody S, Lovel K, Richards D, Gask L, et al. Collaborative care for depression and anxiety problems. Cochrance library. 2012(10). DOI: 10.1002/14651858.CD006525.pub2 PMC11627142 23076925

[10] Reilly S, Planner C, Gask L, Hann M, Knowles S, Druss B, et al. Collaborative care approaches for people with severe mentalillness. Cochrane Database of Systematic Reviews. 2013; 11. DOI: 10.1002/14651858.CD009531.pub2 24190251

[11] Bauer M, McBride L, Williford W, Glick H, Kinosian B, Altshuler L, et al. Collaborative care for bipolar disorder: Part II. Impacton clinical outcome, function, and costs. Psychiatric Services. 2006; 57(7): 937–45. DOI: 10.1176/ps.2006.57.7.937 16816277

[12] Hendrie HC, Lindgren D, Hay D, Lane KA, Gao S, Purnell C, et al. Comorbidity Profile and Health Care Utilization in Elderly Patients with Serious Mental Illnesses. The American journal of geriatric psychiatry. 2013; 21(12). DOI: 10.1016/j.jagp.2013.01.056 PMC3572246 24206938

[13] Siskind D, Harris M, Diminic S, Carstensen G, Robinson G, Whiteford H. Predictors of mental health-related acute service utilisation and treatment costs in the 12 months following an acute psychiatric admission. Aust N Z J Psychiatry. 2014; 48(11). DOI: 10.1177/0004867414543566 25030807

[14] Ose SO, Kalseth J, Ådnanes M, Tveit T, Lilleeng SE. Unplanned admissions to inpatient psychiatric treatment and services received prior to admission. Health Policy. 2018; 122(4). DOI: 10.1016/j.healthpol.2017.12.006 29277424

[15] Winnard D, Papa D, Lee M, Boladuadua S, Russell S, Hallwright S, et al. Populations who have received care for mental health disorders. In: CMDHB (ed.). Auckland: Counties Manukau Health; 2013.

[16] Thornley S, Papa D, Jackson G, Hallwright S. The prevalence and care of mental disorders in Counties Manukau District Health Board from linked health data; 2009.

[17] IHI: Triple aim initiative Institute for Health Care Improvement.

[18] Models of health. Wellington, New Zealand: Ministry of Health; 2016.

[19] Clinical governance: Guidance for health and disability providers. Wellington: Health Quality and Safety Commission New Zealand; 2017.

[20] Kates N, Arroll B, Currie E, Hanlon C, Gask L, Klasen H, et al. Improving collaboration between primary care and mental health services. The World Journal of Biological Psychiatry. 2019; 20(10). DOI: 10.1080/15622975.2018.1471218 29722600

[21] Zanardo G, Silveira L, Rocha C, Rocha K. Psychiatric admission and readmission in a general hospital of Porto Alegre: sociodemographic, clinic, and use of Network for Psychosocial Care characteristics. Revista Brasileira de Epidemiologia. 2017; 20(3). DOI: 10.1590/1980-5497201700030009 29160438

[22] Hamilton J, Heads A, Cho R, Lane S, Soares J. Racial disparities during admission to an academic psychiatric hospital in a large urban area. Comprehensive Psychiatry. 2015; 63. DOI: 10.1016/j.comppsych.2015.08.010 26555499

[23] Bellido-Zanin G, Perez-San-Gregorio M, Martin-Rodriguez A, Vazquez-Morejon A. Social functioning as a predictor of the use of mental health resources in patients with severe mental disorder. Psychiatry Research. 2015; 230(2). DOI: 10.1016/j.psychres.2015.08.037 26343834

[24] Wang J, Chiu C, Yang T, Liu T, Wu C, Chou P. The Low Proportion and Associated Factors of Involuntary Admission in the Psychiatric Emergency Service in Taiwan. PLoS ONE. 2015; 10(6). DOI: 10.1371/journal.pone.0129204 PMC4457903 26046529

[25] Innes H, Lewsey J, Smith D. Predictors of admission and readmission to hospital for major depression: A community cohort study of 52990 individuals. Journal of Affective Disorders. 2015; 183. DOI: 10.1016/j.jad.2015.04.019 25997169

[26] Gerson R, Havens J, Marr M, Storfer-Isser A, Lee M, Rojas MC, et al. Utilization Patterns at a Specialized Children’s Comprehensive Psychiatric Emergency Program. Psychiatric Services. 2017; 68(11). DOI: 10.1176/appi.ps.201600436 28617206

[27] Hamilton JE, Desai PV, Hoot NR, Gearing RE, Jeong S, Meyer TD, et al. Factors Associated With the Likelihood of Hospitalization Following Emergency Department Visits for Behavioral Health Conditions. Academic Emergency Medicine. 2016; 23(11). DOI: 10.1111/acem.13044 27385617

[28] Huntley A, Lasserson D, Wye L, Morris R, Checkland K, England H, et al. Which features of primary care affect unscheduled secondary care use? A systematic review. Bmj Open. 2014; 4. DOI: 10.1136/bmjopen-2013-004746 PMC4039790 24860000

[29] Andersen R. Familys’ use of health service: use of health services: a behavioral model of predisposing, enabling, and need components. West Lafayette, Indiana: Pardue University; 1968.

[30] Anderson R. Revisiting the behavioral model and access to medical care: does it matter? J Health Soc Behav. 1995; 36: 1–10. DOI: 10.2307/2137284 7738325

[31] Anderson R. National health surveys and the behavioral model of health services use. Med care. 2008; 46: 647–53. DOI: 10.1097/MLR.0b013e31817a835d 18580382

[32] Busse R, Stahl J. Integrated care expereinces and outcomes in Germany, the Netherlands, and England. Chronic care. 2014; 33(9). DOI: 10.1377/hlthaff.2014.0419 25201659

[33] Coleman K, Austin BT, Brach C, Wagner EH. Evidence On The Chronic Care Model In The New Millennium. Health affairs. 2009; 28(1): 75–85. DOI: 10.1377/hlthaff.28.1.75 19124857 PMC5091929

[34] Woltmann E, Grogan-Kaylor A, Perron B, Georges H, Kilbourne A, Bauer MS. Comparative Effectiveness of Collaborotive ChronicC are Models for Mental Health Conditions Across Primary, Specialty, and Behavioral Health Care Settings: Systematic Review and Meta-Analysis. American Journal of Psychiatry. 2012; 169. DOI: 10.1176/appi.ajp.2012.11111616 22772364

[35] Render CM, Valk GD, Griffin S, Wagner EH, Eijk JT, Assendelft WJ. Interventions to Improve the Management of Diabetes Mellitus in primary care, outpatient, and community Settings. Cochrane Database of Systematic Reviews; 2002. 10.1002/14651858.CD001481 PMC7045779 11279717

[36] Healthpoint. Counties Manukau Health Wellness Support Model of Care 2019. Available from: https://healthpoint.co.nz/public/community/counties-manukau-health-wellness-support/.

[37] Durie MH. A Maori perspective of health. Social Science & Medicine. 1985; 20: 483–6. DOI: 10.1016/0277-9536(85)90363-6 3992288

[38] Durie MH. Mauri Ora: the dynamics of Māori health. Oxford University Press; 2001.

